# Gene Expression Profiling Reveals Large Regulatory Switches between Succeeding Stipe Stages in *Volvariella volvacea*

**DOI:** 10.1371/journal.pone.0097789

**Published:** 2014-05-27

**Authors:** Yongxin Tao, Arend F. van Peer, Bingzhi Chen, Zhihong Chen, Jian Zhu, Youjin Deng, Yuji Jiang, Shaojie Li, Taju Wu, Baogui Xie

**Affiliations:** 1 College of Horticulture, Fujian Agriculture and Forestry University, Fuzhou, Fujian, China; 2 Mycological Research Center, College of Life Sciences, Fujian Agriculture and Forestry University, Fuzhou, Fujian, China; 3 College of Food Science, Fujian Agriculture and Forestry University, Fuzhou, Fujian, China; 4 State Key Laboratory of Mycology, Institute of Microbiology, Chinese Academy of Sciences, Beijing, China; Macquarie University, Australia

## Abstract

The edible mushroom *Volvariella volvacea* is an important crop in Southeast Asia and is predominantly harvested in the egg stage. One of the main factors that negatively affect its yield and value is the rapid transition from the egg to the elongation stage, which has a decreased commodity value and shelf life. To improve our understanding of the changes during stipe development and the transition from egg to elongation stage in particular, we analyzed gene transcription in stipe tissue of *V. volvacea* using 3′-tag based digital expression profiling. Stipe development turned out to be fairly complex with high numbers of expressed genes, and regulation of stage differences is mediated mainly by changes in expression levels of genes, rather than on/off modulation. Most explicit is the strong up-regulation of cell division from button to egg, and the very strong down-regulation hereof from egg to elongation, that continues in the maturation stage. Button and egg share cell division as means of growth, followed by a major developmental shift towards rapid stipe elongation based on cell extension as demonstrated by inactivation of cell division throughout elongation and maturation. Examination of regulatory genes up-regulated from egg to elongation identified three potential high upstream regulators for this switch. The new insights in stipe dynamics, together with a series of new target genes, will provide a sound base for further studies on the developmental mechanisms of mushroom stipes and the switch from egg to elongation in *V. volvacea* in particular.

## Introduction

**Figure 1:**
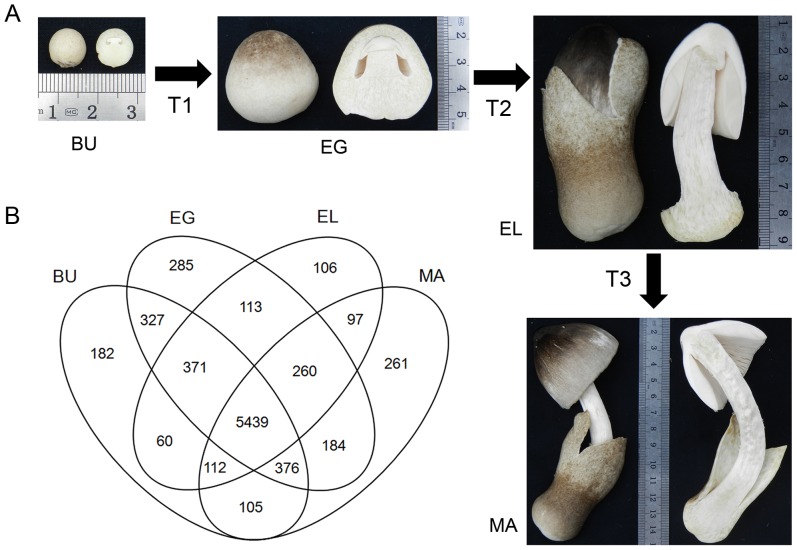
Four developmental stages of *V. volvacea* and corresponding gene expression. (**A**) Showing the four developmental stages of *V. volvacea* as used in this study; BU, EG, EL and MA, and the three intermediate transitions; T1, T2 and T3. Size of the fruiting bodies is indicated in centimeters by the ruler included in the four pictures. The button stage (BU) shows a closed pileus, a ∼0.2 cm long stipe and universal veil. The egg stage (EG) is larger, the universal veil still enveloping the ∼0.5–1 cm long stipe and pileus. The elongation stage (EL) shows a still closed pileus and largely elongated stipe (∼5–7 cm), the ruptured veil will form the volva attached to the bottom of the stipe (better visible in MA stage). The maturation stage (MA) ends with a completely unfolded pileus (picture shows pileus in process of being unfolded) and maximal stipe size of ∼5–10 cm in length and ∼0.5–1.5 cm in diameter. (**B**) Venn diagram showing numbers of expressed genes in stipe tissue of the four depicted stages BU, EG, EL and MA. Each stage has one corresponding circle in the Venn diagram. Overlap between circles and gene numbers herein indicate co-expression of that number of genes between respective stages. For example, the BU stage has 182 uniquely expressed genes, 327 genes that are expressed in the BU stage and the EG stage but not in EL and MA, 371 genes expressed in BU, EG and EL but not in MA, and so on. 5,439 genes are expressed in all four stages, and co-expression between BU and EG (327) is considerably higher than between EG and EL, or EL and MA.

The edible straw mushroom *Volvariella volvacea* is popular in the diets of Southeast Asia and ranks high (5th) in terms of annual world-wide production [1]–[3]. As a rule, *V. volvacea* is harvested in its egg stage, since taste, shelf life, nutritious and thus commodity value are considerably better in this than in later developmental stages [4]. Egg stage fruiting bodies are characterized by a pileus and stipe that are still fully enclosed by the universal veil (exo-pellicle) causing an egg shaped appearance (Figure 1A). According to Chang [5], the egg stage is preceded by the primordium stage (small clusters of hyphae, gradually differentiating in distinct tissues) and the button stage (small spheres, pileus, stipe and other tissues are clearly visible in cross sections). The succeeding stages that follow the egg stage are particularly fast. Rapid stipe extension to near full length in the elongation stage ruptures the exo-pellicle (forming the volva) on average in 5–12 hours, although periods of as little as 3 hours are possible. The elongation stage quickly advances to the maturation stage, with extension of the stipe to its final length, unfolding of the pileus, and lastly sporulation and deterioration.

Little is known about the mechanisms that govern these transitions in mushrooms, and most of our knowledge has been obtained from macro- and microscopic observations. Stipe elongation in general occurs in a short period of time, during which responsiveness to light (phototropism) [6], [7] and gravity (gravitropism) [8], [9] guide the direction of growth. Moreover, extension of stipes is mostly asymmetrical, with considerably stronger extension of the mid to upper part of the stipe (near the pileus) than of the basal section [10]–[14]. A presumed role herein for growth factors emanating from the pileus remains to be resolved yet, as experiments analyzing decapitation at various stages of stipe elongation are contradicting [10], [15]–[17].

Furthermore, two morphologically distinct types of hyphae are recognized within a developing stipe, inflated hyphae and narrow hyphae. The elongation of stipes has been attributed to further inflation of the inflated hyphae, chiefly in vertical direction. This is in contrast to extensive cell division as a driving force of tissue expansion [10], [14], [17]–[19]. Narrow hyphae are unknown to drive elongation directly, but the ratio of inflated hyphae increases over the course of elongation, suggesting differentiation of at least a part of the narrow hyphae into inflated hyphae [17]. In *Coprinus cinerea*, inflated hyphae have been reported to be multinucleated, and accumulation of nuclei is thought to occur before rapid elongation [17], [20].

Molecular genetic studies have started to reveal some of the genes involved in the processes of mushroom stipe elongation. Fast extension of the stipe is accompanied by high activity of chitin synthases during elongation [21] and autolysis of (β) glucans that confer rigidity and strength to fungal cell walls [13], [22]–[24]. Studies on *exg2* of the mushroom *Lentinula edodes* identified a glycoside hydrolase family 55 (GH55) exo-β-1,3-glucanase with a function in stipe elongation [25], [26] and we recently reported possible involvement of *exg2* in stipe elongation in *V. volvacea* [27]. To this, a collection of *C. cinerea* “elongationless” mutants (which fail to elongate stipes) suggests involvement of at least eight different genes (*eln1* to *eln8*) of which three have been cloned and identified. Genes *eln2*, *eln3* and *Cc.cdc3* (*eln8*), encoding a cytochrome P450, a putative glycosyltransferase and a septin protein, all are essential for proper stipe elongation [28]–[30]. However, the regulation of those genes, as well as genetic regulation of any other process during stipe elongation remains to be established.

Development of stipes, elevating the pileus to facilitate spore dispersion, is a principal step in the life cycle of most (Agaric) mushrooms and better comprehension of the underlying mechanisms is essential to understand these important organisms. In our particular case, it will help to identify the control mechanisms of the swift transition from valued eggs to less tasty and deteriorating elongation and maturation stages, a central problem for cultivation and production of *V. volvacea*. We performed global gene expression profiling of stipes of four developmental stages in order to provide a first genetic framework. Our data demonstrates clearly distinct regulation of cell cycle and metabolic processes in different stages, and we identified a substantial set of regulatory genes.

## Materials and Methods

### Organisms and growth condition

*V. volvacea* H1521 was obtained from the Agricultural Culture Collection of China (ACCC52633), and maintained with periodic transfers on potato dextrose agar, at 20°C. For cultures producing fruiting bodies, strain H1521 was cultivated on rice straw compost according to Chen [31]. Samples for RNA isolation were collected from multiple stipes of *V. volvacea* at four typical developmental stages: button stage (BU, day 10 after inoculation), egg stage (EG, day 13 after inoculation), elongation stage (EL, day 13.5 after inoculation) and maturation stage (MA, day 14 after inoculation). Stipes from the BU stage were excised from over 100 separate fruiting bodies (totaling ∼5 grams). Stipes from the EG stage totaled about 10 grams derived from 50 fruiting bodies. For the EL and MA stages, stipes of 25 fruiting bodies were collected (more than 100 grams each sample). Stipes of a designated stage were excised, chopped, mixed, and divided in small portions and frozen in liquid nitrogen. For expression pattern verification of individual genes (quantitative PCR), new batches of H1521 fruiting bodies were grown and sampled according to the described method.

### RNA sample preparation

Total RNA was extracted using pBIOZOL Plant Total RNA Extraction Reagent (BioFlux, China) according to the manufacturer's protocol, followed by purification with an RNeasy plant mini kit (QIAGEN, Germany) to remove potential genomic DNA. RNA quality and concentration were evaluated using an Agilent 2100 Bioanalyzer (Agilent Technologies, Palo Alto, CA, USA).

### cDNA library preparation and sequencing

A total of 6 µg of RNA from each sampled stage was used for cDNA library construction. Poly (A) mRNA was enriched with oligo (dT) magnetic beads and used as template for cDNA synthesis using oligo (dT) as primer. The ds-cDNAs were digested by restriction enzyme NlaIII to generate the CATG sites and subsequently digested by restriction enzyme Mme I which cuts 17 bp downstream of the CATG site, acquiring tags with different adaptors of both ends to form a tag library. After linear PCR amplification and purification, the library was sequenced using Illumina HiSeq 2000 at Beijing Genome Institute (BGI, Shenzhen, China).

### Data processing

After removal of empty reads with only adaptor sequence and poor quality tags (low sequence quality / too long or too short / with only one copy), clean tags were mapped to the predicted genome of *V. volvacea*, allowing one base mismatch. Ambiguous tags were excluded. Gene expression was measured by counting of the number of unambiguous clean tags for each gene and normalized to number of transcripts per million clean tags (TPM) [32], [33].

### Screening of differentially expressed genes (DEGs)

Fold change of gene expression in combination with false discovery rate (FDR) control was used to distinguish differentially expressed genes between samples. FDR was used to determine the threshold of the P-value (corresponding to a differential gene expression test) in multiple tests and analyses. Our analysis used “FDR ≤0.001, and an absolute log2-fold change ≥1” [34] as thresholds to judge the significance of the gene expression difference.

### Quantitative PCR (Q-PCR) verification

Total RNA was isolated from stipes of four developmental stages of *V. volvacea* (see above) using the RNAprep Pure Plant Kit (TIANGEN, China) according to the manufacturer's protocol. Extracted RNA was quantified using an ND 1000 Spectrophotometer (Nano Drop Technologies, USA). Only RNA samples with A260/A280 ratios between 1.9 and 2.1 and A260/A230 ratios greater than 2.0 were used for further analysis. cDNA was synthesized with random primers and oligo dT primer using the PrimeScript RT reagent Kit (TAKARA, Japan) according to the manufacturer's protocol.

Primers (Table S1) were designed across introns using DNAMAN 6.0, based on gene structures according to the genome sequence and matches with transcriptome raw reads obtained by Illumina sequence using ZOOM software [35]. Q-PCR was performed using a CFX96 Real-Time PCR Detection System (BIO-RAD, USA) with SsoAdvanced SYBR Green Supermix (BIO-RAD, USA). Reactions followed denaturation for 10 s at 95°C, 40 cycles of 5 s at 95°C and 30 s at primer-specific annealing temperatures. The glyceraldehyde-3-phosphate dehydrogenase gene (GAPDH) was used as an internal control gene. The 2^-ΔΔCt^ method was used for Q-PCR data analysis [36].

### Sequence accession

The draft genome of *V. volvacea* PYd21 (ACCC52632) is available under accession no. PRJNA171553 at NCBI. Raw digital gene expression profiles of stipe tissue stages of *V. volvacea* fruiting bodies are deposited at GEO database: GSM1060233-GSM1060236, http://www.ncbi.nlm.nih.gov/geo/query/acc.cgi?token=pxcbpqmmwqeuopo&acc=GSE43297.

## Results

### Digital gene expression profiling of stipe tissue

Digital gene expression (DGE) analysis was performed for *V. volvacea* stipe tissue from four distinct developmental stages: button (BU), egg (EG), elongation (EL) and maturation (MA) (Figure 1A). The proportion of clean tags on the total of acquired tags was > 97% showing high sequencing quality. Comparable numbers of clean tags and unique tags were obtained for the respective different stages (Table S2). Expression profile sequencing saturation analysis showed that the number of genes represented by clean tags stabilized when the number of total tags reached 2 million or higher (Figure S1). Obtained tags (> 5 million) therefore represented full coverage for each sample.

After mapping of unambiguous clean tags against 11,534 predicted genes of the *V. volvacea* draft genome, 8,278 genes (71.7% of the genome) were found to be expressed in at least one stage, of which 5,439 genes (47.2% of the genome, 65.7% of expressed genes) were expressed in all four stages (Figure 1B, Table S3). The highest number of expressed genes was found in stipes of the EG stage with 7,355 genes (63.8% of the genome, 88.8% of expressed genes), followed by the BU stage with 6,972 genes (60.4%, 84.2%), MA stage with 6,834 genes (59.3%, 82.5%) and EL stage with 6,558 genes (56.9%, 79.2%), (Table S2, S3). Comparison of expressed genes between different stages revealed that the EG and MA stage contained the highest number of stage specifically expressed genes while the EL and BU stage contained considerable lower numbers (Figure 1B, Table S3). Co-expression of succeeding stages was particularly strong between the BU and EG stage, and almost three times higher than between EG and EL or EL and MA (Figure 1B, Table S3).

To explore whether gene expression of the four stipe stages reflected distinguishable molecular biological processes, we performed COG and KEGG annotation and compared the profiles of each stage. Patterns for designated functional terms were basically uniform between the four stipe stages, and no clear indication of stage specific processes could be derived from this (Table S4A, S4B). Analysis of COG and KEGG patterns for the stage specifically expressed genes (uniquely expressed, BU: 182; EG: 285; EL: 106; MA: 261 in Figure 1B) did show larger enrichment of COG terms: L: “Replication, recombination and repair”, K: “Transcription”, E: “Amino acid transport and metabolism” and T: “Signal transduction mechanisms” and KEGG term “Replication and repair” in the EG stage (Table S5A, S5B). However, both numbers and expression levels of specifically expressed genes were low, revealing limited information (Figure 1B, Table S3, S5).

### Analysis of DEGs between successive stipe stages

**Figure 2:**
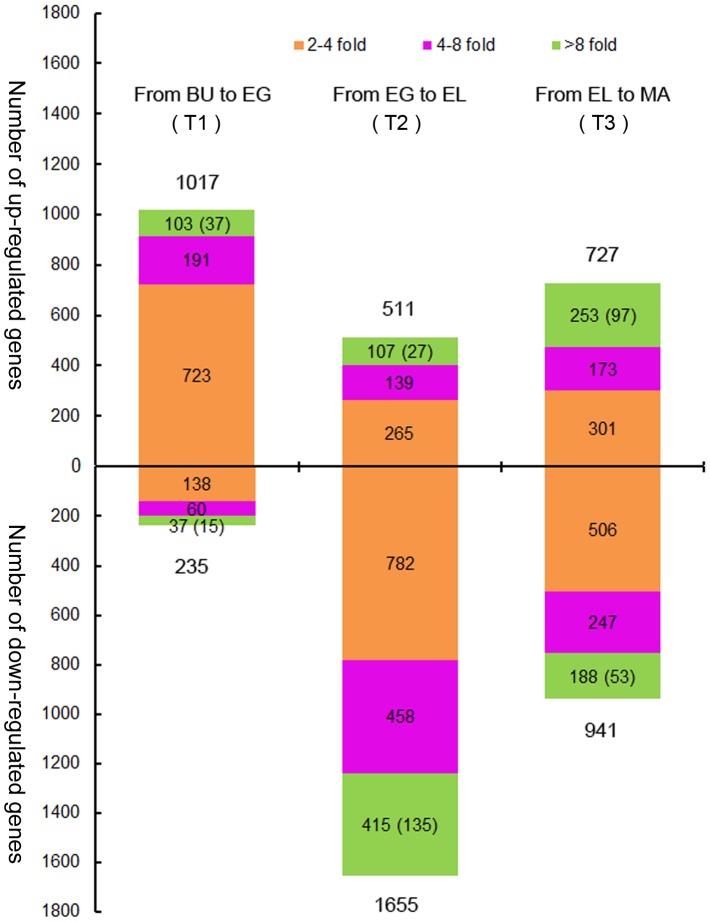
Changes in gene expression between succeeding stages; transitions T1, T2 and T3. Differentially expressed genes (DEGs) are divided over three groups of absolute log2 fold changes in expression; 2 to 4 fold (orange), 4 to 8 fold (purple) or 8 or more fold (green). Genes with expression in only one of two compared stages (thus uniquely expressed in that particular comparison) predominantly show >8 fold changes and are additionally indicated between brackets within this group. Considerably more genes are up-regulated from BU to EG, whereas more genes are down-regulated from EG to EL. Numbers of up- and down-regulated genes from EL to MA are more balanced, although more genes are down- than up-regulated. In each transition, the largest number of DEGs is represented by the 2–4 fold group (orange). Relative high numbers of genes are completely turned off from EG to EL (135) and turned on from EL to MA (97).

To increase the resolution of functional transitions during stipe development, we then focused on expression level changes of genes between successive stages: BU to EG, EG to EL and EL to MA (Figure 1, 2, Table S6). Changes were classified according to absolute log2-fold changes in up- or down-regulation; less than 2 times, 2 to 4 times, 4 to 8 times, and 8 or more times. Genes with small changes in expression (absolute log2-fold <1) were discarded. 2 to 4 fold changes constituted the largest class of DEGs in each transition, indicating a significant role of temperate expression level changes during regulation of stipe development. DEGs with 4–8 fold or more than 8 fold changes contributed to a varying extent to gene expression profiles. Genes with no expression in one of two compared stages (commonly >8 times up- or down-regulated) were relatively sparse, showing a lesser role for gene on/off based regulation (Figure 2, indicated in brackets).

The first transition (BU-EG) involved the lowest total number of DEGs (1,252, Table S6A) and showed up-regulation of more than 81% of the DEGs, predominantly mediated by 2–4 fold up-regulation (71.1 % of up-regulated genes). Contribution of 4–8 fold and 8 or more fold expression changes were small in this transition, and very few genes were turned on or off (37 respectively 15). The next transition (EG-EL) was accompanied by the highest number of DEGs (2,166, Table S6B), of which more than 76% showed down-regulation. Moreover, considerably higher percentages of genes were down-regulated 4–8 and 8 or more fold (together 40.3% of the DEGs) and 135 DEGs were turned off completely. Thus, a general and strong down-regulation of most genes accompanied this transition. The last transition (EL-MA) showed altered expression of 1,668 genes (Table S6C) with slightly more overall down-regulation (56.4% down, 43.6% up). A third of the up-regulated genes showed 8 or more fold up-regulation, of which 97 were turned on, indicating the start of a series of new processes after the strong down-regulation between EG-EL.

**Figure 3:**
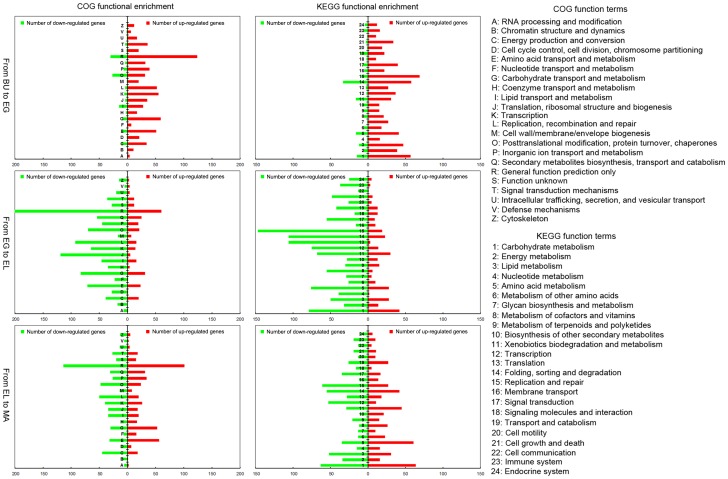
COG and KEGG based functional annotation of differentially expressed genes. Clear changes in regulation of biological functions are shown. The most important changes are: almost exclusive up-regulation of DNA replication and cell cycle related processes from BU to EG, down-regulation of those processes from EG to EL, and up-regulation of transport related processes from EL to MA (even though more genes are down- than up-regulated during transition from EL to MA).

22 COG terms and 24 KEGG pathway subgroups (8 KEGG groups with less than 4 genes in all three stage-transitions together were excluded) associated to the DEGs showed more up-regulated genes from BU to EG, more down-regulated genes from EG to EL, and balanced numbers of up- and down-regulated genes from EL to MA (Figure 3). However, COG terms associated with DNA / RNA replication and processing (COG term: A, B, F, H, J, K, L) or cell division (COG term: D, Z) almost exclusively contained up-regulated genes from BU to EG and down-regulated genes from EG to EL (Figure 3). Related KEGG terms in BU-EG and EG-EL (nucleotide and cofactor / vitamin metabolism 4, 8; transcription, translation and replication, 12, 13, 15; cell growth and death, 21) showed a similar deviation, suggesting specific up- followed by down-regulation of the cell cycle and or cell division from BU to EG to EL. Interestingly, COG and KEGG terms associated with metabolic activity and transport showed disproportional numbers of up-regulated genes in the transition from EG-EL, when compared to the number of all COG / KEGG term up-regulated genes in this transition. In the transition from EL to MA, more DEGs associated to COG and KEGG terms relating to cell cycle or cell division processes were down-regulated as well, while COG and KEGG terms relating to transport and metabolism mostly showed higher numbers of up-regulated genes than down-regulated genes (COG term: E, G, H and to a lesser extend I and Q; KEGG term: 1, 4, 5, 6, 8, 11, 14, 19). Together, this suggested a very large developmental shift between EG and EL especially targeting the cell cycle, that is continued from EL-MA. In addition, the shift encompasses the activation of metabolic and transport pathways from EG to EL and EL to MA.

### Cell cycle and cell division in the developing stipe

**Figure 4:**
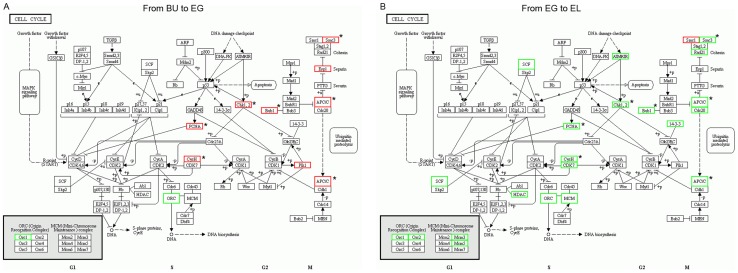
Differentially expressed genes associated to the cell cycle pathway in transition T1 and T2. (**A**) DEGs between BU and EG, associated to the KEGG cell cycle pathway in the KEGG database. All except one gene are up-regulated. Up-regulated genes are boxed red, down-regulated genes are boxed green. Asterisks indicate genes for which DGE data was verified by relative Q-PCR (Table S7B). (**B**) DEGs between EG and EL, associated to the KEGG cell cycle pathway in the KEGG database. All except one gene are down-regulated. Up-regulated genes are boxed red, down-regulated genes are boxed green. Asterisks indicate genes for which DGE data was verified by relative Q-PCR (Table S7B).

In order to verify up- and down-regulation of the cell cycle and cell division during the transitions from BU to EG and EG to EL, we analyzed the KEGG pathway “cell cycle” (KEGG “Cellular processes / cell growth and death / cell cycle”) in more detail. Twelve genes in this pathway were found to be differentially expressed during the transition from BU to EG, and eleven of these were up-regulated (Figure 4, Table S7A). Twenty one DEGs matched this pathway during transition from EG to EL, of which twenty were down-regulated. Six cell cycle genes were found to be differentially expressed between EL and MA, two showed up- and four showed down-regulation (Figure S2). Seven selected KEGG cell cycle pathway genes showing differential expression from BU to EG as well as from EG to EL (asterisk, Figure 4 and Table S7A, S7B) were verified by Q-PCR and confirmed the DGE data. Cell cycle controlling genes G1 cyclin, G1-S cyclin, G1 Cdk (Cyclin-dependent kinase), G1-S Cdk, S cyclin and the G1-S transcription factor (homologues based on *Saccharomyces cerevisiae*) [37] showed up-regulation from BU to EG and down-regulation from EG to EL as well (Table S8A, DGE and Q-PCR). Finally, five selected mitotic kinases that regulate cytokinesis (Plo1p, Cdc5p, Cdc7p, Cdc14p and Mob1p, *Schizosaccharomyces pombe*) [38] showed up- and down-regulation corresponding to the KEGG cell cycle pathway and cell cycle regulator patterns (Table S8B, except Mob1p which was not up-regulated from BU to EG). Clearly, cell division is up-regulated from BU to EG, and strongly reduced during transition to the EL stage in *V. volvacea*. From EL to MA, the majority of cell cycle genes and regulators remain or are even further down-regulated (Figure S2 and Table S7, S8).

### Regulatory factors of stipe elongation in *V. volvacea*

Expression data was screened for predicted regulators involved in the switch from EG to EL. Twenty-four predicted regulatory factors were up-regulated from EG to EL in *V. volvacea* (Table S9, Data S1), and up-regulation from EG to EL was confirmed by Q-PCR for twenty-one of these genes (Table 1). Of ten predicted transcription factors, three genes are likely to function highly upstream in regulation of EG to EL transition: two jumonji family transcription factors (chromatin regulation) and a transcription factor containing a bromodomain (chromatin-associated, histone acetyltranferase). Other important regulators comprised a SART-1 family transcription factor (cell cycle arrest involved), two serine/threonine protein kinases (signal transduction and proliferation) and a thioredoxin (putative regulation of growth factors).

**Table 1:** Predicted regulatory factors of *V. volvacea* that are up-regulated in stipe tissue from EG to EL.

Gene ID	Protein name or domain	Known function in fungi	Reference	Expression level in EL stage (TPM)	Log2 fold changes (EL/EG)
GME9038_g	Serine/threonine protein kinase	Important mediators of fungal proliferation and development as well as signal transduction and infection-related morphogenesis	[44]	1914.78	1.69
GME10647_g	Cytochrome P450	Involved in stipe elongation	[28]	1460.70	3.82
GME5859_g	Protein prenyltransferase alpha subunit repeat	Regulating cellular process: involved in signal transduction and intracellular vesicle transport	[45]	1301.47	1.82
GME7934_g	JmjC domain, jumonji family of transcription factors	Participating in negative regulation of cell proliferation signaling	[42], [43]	206.61	1.35
GME9386_g	Thioredoxin	Redox regulation of growth factors, cytokines, transcription factors involved in growth, differentiation	[46]	197.91	3.25
GME8711_g	Fungal specific transcription factor; GAL4-like Zn2Cys6 DNA-binding domain	Regulating a variety of cellular and metabolic processes	[47], [48]	192.05	1.80
GME8678_g	bZIP transcription factor	Regulating a diverse set of cellular pathways	–	125.58	1.01
GME1797_g	Phytochrome-like protein	Possibly involved in reproduction and spore formation	[49]	123.73	2.04
GME10493_g	Two-component sensor protein, histidine protein kinase	Responding to environmental stimuli and regulating developmental pathway	[50]–[52]	78.69	1.61
GME9838_g	Jmj domain, jumonji family of transcription factors	Participating in negative regulation of cell proliferation signaling	[42], [43]	75.68	1.58
GME9978_g	Chitin-binding domain	Participating in metabolism of chitin in fungal cell walls	[26], [53]	58.43	1.23
GME6807_g	SART-1 family transcription factor	Involved in cell cycle arrest and pre-mRNA splicing	[54]	37.51	1.14
GME9454_g	Serine/threonine kinases, fungal mitogen-activated protein kinases Sty1 and Hog1 subfamily, catalytic domain	Participating in controlling intracellular events including responses to hormones and major developmental changes and involved in cell wall construction and morphogenesis	[55], [56]	33.15	1.72
GME10889_g	bZIP transcription factor	Regulating a diverse set of cellular pathways	–	22.44	2.02
GME8409_g	SANT/myb-like domain protein	Unknown	–	16.74	1.60
GME8831_g	GAL4-like Zn2Cys6 binuclear cluster DNA-binding domain, fungal specific transcription factor	Regulating a variety of cellular and metabolic processes	[47], [48]	14.57	1.74
GME1644_g	GATA zinc finger DNA binding domain, transcription factor	Regulating nitrogen metabolism, light induction, siderophore biosynthesis and mating-type switching	[57]	13.90	1.13
GME4506_g	Bromodomain transcription factor	Regulating in histone-directed chromatin remodeling and gene transcription	[41]	11.89	1.83
GME1998_g	MADS (MCM1, Agamous, Deficiens, and SRF) box family of eukaryotic transcriptional regulators	Developmental control and signal transduction	[58]	10.05	1.58
GME1283_g	DHHC palmitoyltransferase, ankyrin repeats	Unknown	–	6.20	2.21
GME3988_g	GH3 auxin-responsive promoter, indole-3-acetic acid-amido synthetase	Related to growth and development	–	4.86	8.92

Analysis of orthologs of stipe elongation genes previously identified in *Coprinopsis cinerea* (*eln2*; cytochrome P450, *eln3*; glycosyltransferase, *eln8; Cc.Cdc3*) confirmed up-regulation of those genes in the EL stage in *V. volvacea*, although the *eln2* and *eln3* orthologs were lowly expressed (Table S10). Interestingly, another cytochrome P450 (GME10647_g, Table 1) showed very high expression and specific up-regulation in the EL stage, suggesting involvement of multiple P450s. Finally, one gene likely to be involved in cell-wall modulation was found included within the potential regulators (CBM family 12; chitin binding, Table 1), fitting well with the general understanding that stipe elongation requires extensive reorganization of cell walls.

## Discussion

Elongation of stipes is an important event during the general life cycle of many mushrooms, and bears significant economic implications for most commercially cultivated species. However, our understanding of this process is minimal, and molecular genetic information on its regulation is virtually absent. In this study we decided to focus on total gene expression profiles of developing stipes from the mushroom *V. volvacea*, to generate a first genetic framework for stipe development and to identify a series of putative regulators of stipe elongation. Samples were taken from stipe tissue of four classically separated, succeeding stages of *V. volvacea*; button (BU), egg (EG), elongation (EL) and maturation (MA). The obtained expression profiles reached saturation for all four stages as described in the results (see Figure S1) and although annotation of mushroom genomes is complicated by gaps in knowledge on protein functions, we established an average association of >56% of the genes with COG and >61% of the genes with KEGG terms (see Table S4). Together, this enabled us to identify main functional switches in *V. volvacea* stipe development.

Despite its superficial simplicity, the developing stipe of *V. volvacea* undergoes a complex series of changes indicated by vast numbers of expressed genes in each individual stage (Table S2, S3) and large groups of genes with alterations in expression levels (Figure 2). On/off based regulation of functional processes in the stipe of *V. volvacea* is of minor importance, as is evident from low numbers of stage specific expressed genes (Figure 1), very low expression of stage specific genes (Tables S3), and from the low numbers of on/off regulation of DEGs between successive stages (Figure 2, Table S6). Accordingly, functional annotation of stage specific expressed genes revealed little functional differences between stages. Differences between succeeding stages became much clearer when regarding changes in gene expression. The largest numbers of DEGs was made up by genes changing 2-4 fold, followed by either 4–8 or 8 or more fold changes in expression (depending on the respective transition). Regulation of stipe development therefore appears to be largely mediated by (medium) changes in gene expression levels, instead of through absolute (on/off) expression of genes.

The first transition, from BU to EG, is that of the second most complex stage to that of the most complex stage in terms of numbers of stipe expressed genes (Table S2; 6,972 and 7,355), suggesting larger variety of processes in those stages than in the EL and MA stage. However, differences in functional processes between the BU and EG stage seem to be smaller than between EG-EL and EL-MA. BU and EG share clearly more co-expressed genes (Figure 1B) and the number of total DEGs (1,017+235 = 1,252) is lower than in either transition T2 (2,166) or T3 (1,668). Over 68% of the DEGs involved changes within 2-4 fold, the majority being up-regulated, while very low numbers of down-regulated genes were found for any functional group. Complete on/off regulation of DEGs was very small as well (37 and 15 genes respectively), indicating very few new or aborted processes. Most of the existing processes are likely to be maintained or increased, but probably not reduced or stopped. The clearest functional difference between BU and EG was the exclusive up-regulation of cell-cycle processes and cell division (no down-regulation), which implies an important role for cell division as means of growth during development of the stipe from BU to EG (Figure 1, see size differences between the stipe in the BU and the EG stage).

The second transition (T2) involved that from the most complex to the least complex stipe stage in terms of expressed genes (EG to EL), and contained the highest number of DEGs (2,166). The process is characterized by overall and particularly strong down-regulation; more than 76 % of all DEGs are down-regulated and more than 40% of all DEGs are down-regulated 4–8 or 8 or more fold. In comparison, 4–8 fold together with 8 or more fold down-regulation constituted 7.7% in T1, and 26.1% in T3. Down-regulation affected all processes, yet in particular targeted the cell-cycle and cell division related functions which showed no up-regulation. Subsequent Q-PCR analysis of the cell cycle and cell division confirmed that these processes were indeed strongly down-regulated. Metabolism and transport related functions showed (next to high numbers of down-regulated genes) the highest proportion of up-regulated genes in transition from EG to EL. Distinct metabolic and transport pathways are thus important (up-regulation) during rapid elongation, although correlation with transport of nutrients to the pileus must be considered as well. Our data further confirms earlier macro- and microscopic observations on developing stipes on the genetic level. Measurements on *V. volvacea* showed conservation of cell size in stipes between BU and EG, whereas elongation of cells (up to 5.8 times) was observed in the EL stage [39]. Similar findings have been reported for *Agaricus bisporus* [40] and for the model mushroom *C. cinerea* [17], [20]. Thus, stipe growth from BU to EG is mainly a result of cell division, confirmed by our data showing up-regulation of cell-division genes, while rapid stipe extension is the result of stipe cell elongation, confirmed by strong down-regulation of cell division (and thus excluding the possibility of cell division as main source of growth). It remains a very interesting question whether the down-regulation of cell division is entirely necessary for cell elongation, or merely beneficial, saving energy.

The third and last transition from EL to MA is accompanied by many functional changes as can be observed from considerable up- as well as down-regulation of genes within most annotated functional groups. A relatively large number of DEGs is turned on (97) or strongly up-regulated indicating activation of processes other than occurring in the EL stage, but cell cycle and cell division remain or are even further down-regulated as was shown by DGE and Q-PCR analysis. The strongest increases are found in metabolism and transport processes, just as between the EG and EL stage. Together, continued down-regulation of the cell cycle, and continued up-regulation of metabolic and transport processes (whether or not exactly the same as in the EL stage) suggest a kind of overall shift that starts in the EL stage and continues in the MA stage.

Very roughly, the change of the stipe from BU to EG could be seen as a continuation of the BU yet with higher activity, followed by a major developmental shift towards the EL that is continued throughout the MA. The analysis of regulatory genes that control this major switch was very promising. Chromosomal changes underlie large developmental shifts, and at least three specifically up-regulated chromatin influencing transcription factors were found. GME4506_g has a predicted bromodomain, strongly associated with histone acyltransferases [41] which in turn control activation of gene transcription. Moreover, two jumonji transcription factors (GME7934_g and GME9838_g) were found. The function of these has been implied in histone demethylation, as well as in control of cell cycle events (amongst others via cyclin D1) [42], [43], and strong regulatory changes in the cell cycle appear to be one of the major characteristics of the EG-EL switch. Identification of yet another transcription factor with a possible function in again the cell cycle (SART-1 domain, GME6807_g) further accredits the significance of the identified regulators for the EG-EL shift. The identified regulators will be our primary targets for further elucidation of the stipe elongation regulatory network.

## Supporting Information

Figure S1 **Gene expression profile saturation analysis.**
(TIF)

Figure S2 **Genes differentially expressed between EL and MA associated to the cell cycle pathway.**
(TIF)

Table S1 Sequences of primers used for Q-PCR.
(DOCX)

Table S2 Summary of the statistics of the digital gene expression data.
(DOCX)

Table S3 Genes expressed in stipe tissue of one or more stages.
(XLSX)

Table S4 COG and KEGG pathway annotation of all expressed genes.
(XLSX)

Table S5 COG and KEGG pathway annotation of stage specifically expressed genes.
(XLSX)

Table S6 Genes differentially expressed between successive stipe stages.
(XLSX)

Table S7 Differential expression of cell cycle pathway genes between successive stipe stages.
(XLSX)

Table S8 Expression levels of genes regulating the cell cycle and cytokinesis.
(XLSX)

Table S9 Digital gene expression levels and relative Q-PCR expression levels of regulatory genes up-regulated from egg stage to elongation stage.
(XLSX)

Table S10 *V. volvacea* orthologs of *C. cinerea* stipe elongation genes.
(XLSX)

Data S1 **Amino acid sequences of predicted** ***V. volvacea*** **genes as used in this study.**
(TXT)
