# Supplementary figures and images for: Gene Expression Profiling Reveals Large Regulatory Switches between Succeeding Stipe Stages in Volvariella volvacea
Source: PLoS One. 2014 May 27;9(5):e97789. doi: 10.1371/journal.pone.0097789 (PMC4035324; doi:10.1371/journal.pone.0097789)

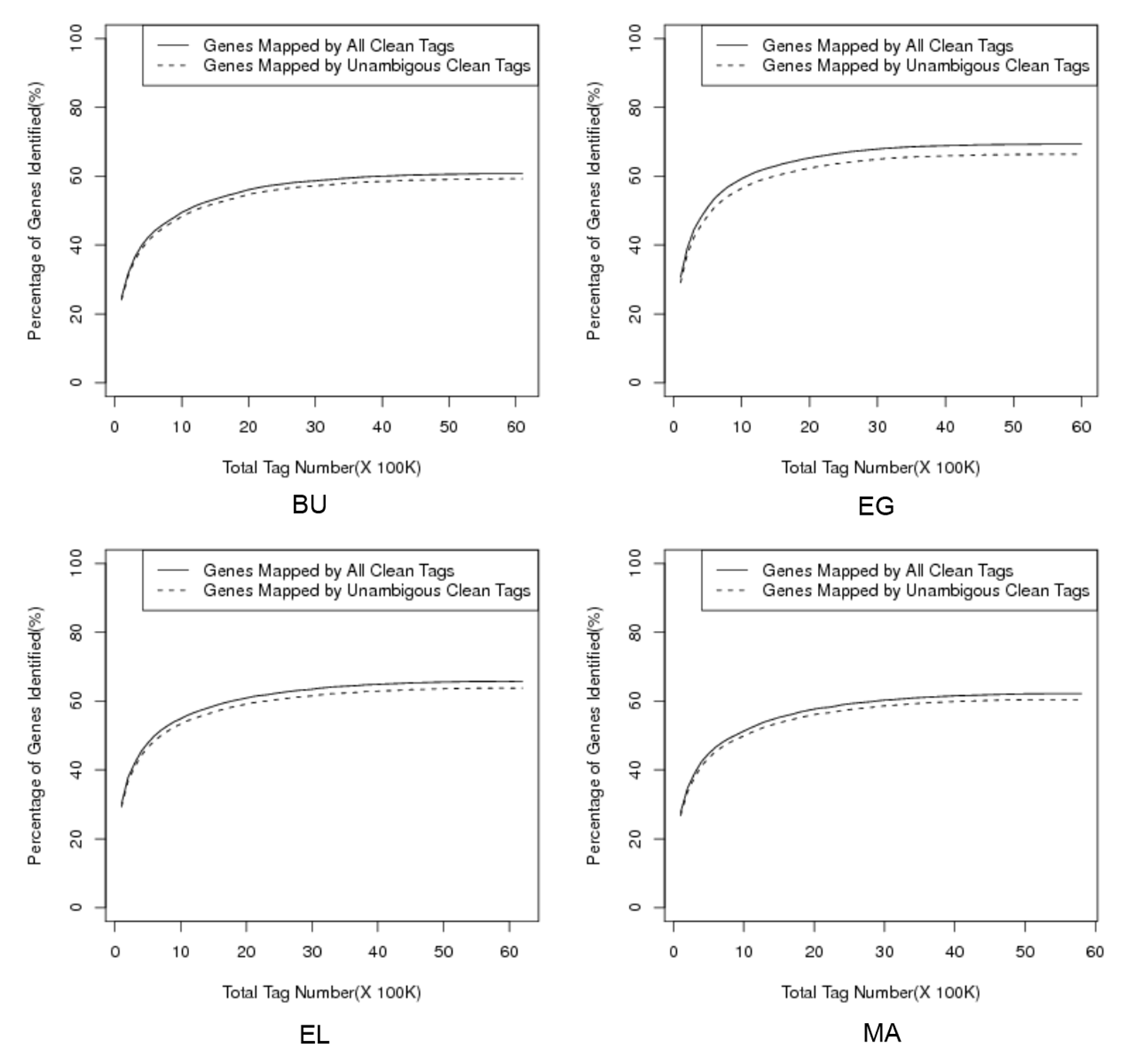

Supplement: Figure S1 — Gene expression profile saturation analysis. (TIF) [file pone.0097789.s001.tif]

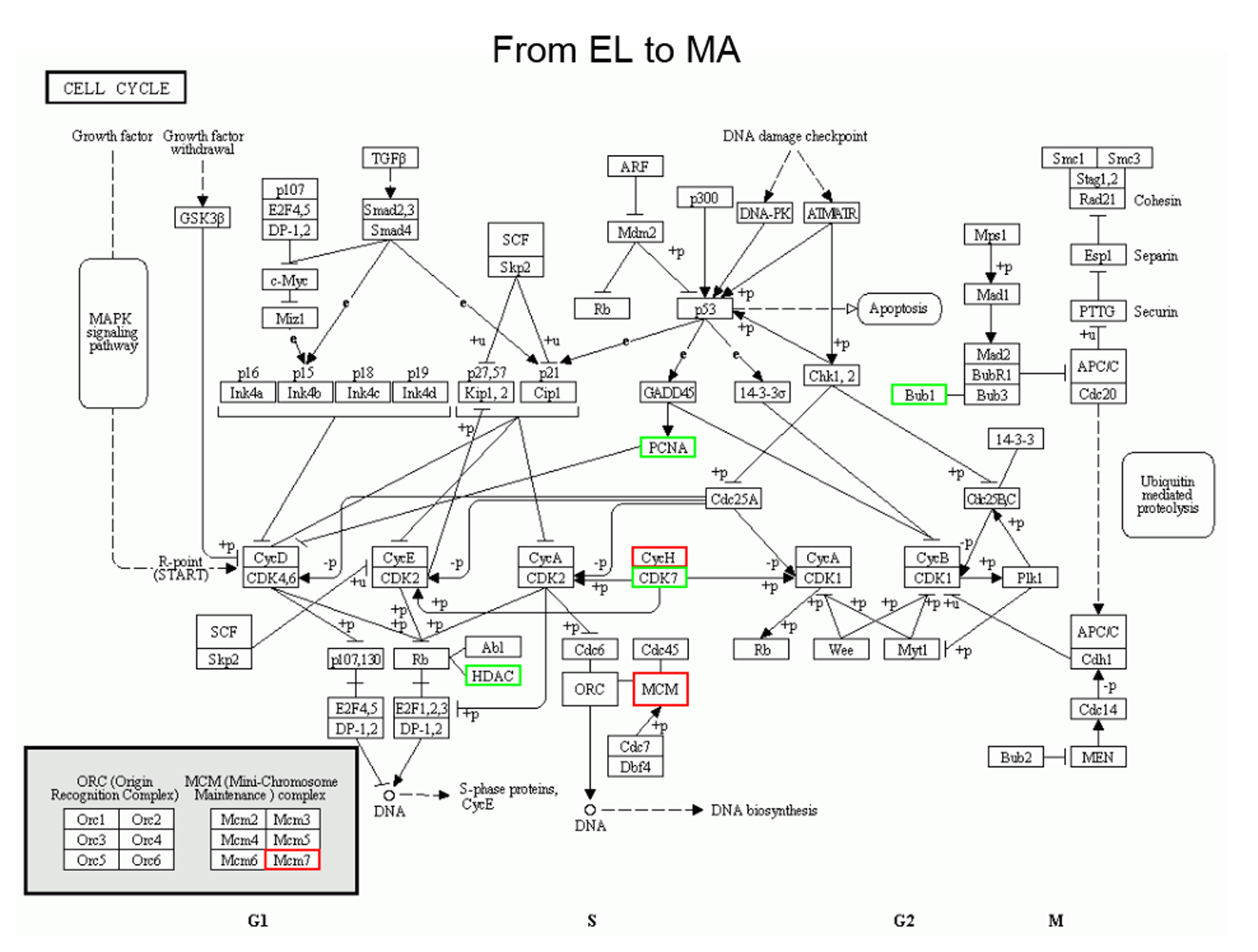

Supplement: Figure S2 — Genes differentially expressed between EL and MA associated to the cell cycle pathway. (TIF) [file pone.0097789.s002.tif]
