# Supplementary material for: Gene Expression Profiling Reveals Large Regulatory Switches between Succeeding Stipe Stages in Volvariella volvacea
Source: PLoS One. 2014 May 27;9(5):e97789. doi: 10.1371/journal.pone.0097789 (PMC4035324; doi:10.1371/journal.pone.0097789)
Supplement: Table S1 — Sequences of primers used for Q-PCR. (DOCX) [file pone.0097789.s003.docx]

Table S1. Sequences of primers used for Q-PCR

| Gene ID | Primer sequences  (5’–3’) | Product size (bp) | Annealing temperature (°C) |
| --- | --- | --- | --- |
| GME10808_g | F: TTGCTCGGATGGGTGGATATTC  R: CTTCAAGAAGTTCATCGGTCGTC | 138 | 60.2 |
| GME4497_g | F: CGCATATACAGTGTTATGGGAAAC  R: CGACTCTACTGTACTCTTCCCAC | 239 | 59.5 |
| GME2815_g | F: GGGAGGTGTTGTAATTGAGATG  R: GGAACATCGTTACTCATCATCAGC | 126 | 59.0 |
| GME6224_g | F: GAGAGACCCTGAGATTTACGCTG  R: TCAAATCGTGCTTGGCTGTTATC | 108 | 60.2 |
| GME11160_g | F: TACCACCATCCACCCCAGCAGT  R: CCTCAGATTCCGATGCAGACACT | 198 | 62.1 |
| GME3484_g | F: CAGTTCATTACGACGACCTTCAG  R: TCTACGAACTCCATAGCTTCCTC | 131 | 60.2 |
| GME11461_g | F: CAGAAAAGGTGGTAGGGAGTAAG  R: AATCGACTAGGCCAGCTCTCTTG | 171 | 61.0 |
| GME9953_g | F: CGCCGAATACCAGATGCAAATACC  R: CCTCCACATCTCCTATCTCATCC | 185 | 61.7 |
| GME11480_g | F: CAGCGATTCTTTCCCTAGTTCAC  R: GCGAGACGTTGTTCAAATTATGC | 170 | 59.2 |
| GME2873_g | F: TGGTCGGTCGGGTGCATATTC  R: ATCCAATCCCAGCTCATCTAATGC | 228 | 61.0 |
| GME10202_g | F: CGTTGATGAAGAAGCGGCTAAGC  R: ATCACGGACCTTTTGCATGAACC | 203 | 60.9 |
| GME3132_g | F: GTCTTCGGTCTGGCTGCTGATG  R: CGCATTCAAAAGGATCGGGTGTTC | 140 | 62.1 |
| GME10647_g | F: CTTGATAGGGGAAGAGTAGTGC  R: CCTTCATGTCCTTCTCCCACTG | 187 | 60.9 |
| GME5148_g | F: GGGTTCCACTGGTAAAGAAGTCG  R: ACCATCATTACGATACACAAGAAGC | 114 | 59.5 |
| GME11273_g | F: GTCTTCCAACAAAAGGTCCAGG  R: TCGCTTCTTCTCTTCCAACTCG | 129 | 59.6 |
| GME9038_g | F: TTAGGAGGTGCGACTGGTGGAT  R: TTCTTCCAAACACGCTGAATCACC | 212 | 61.5 |
| GME5859_g | F: GAGTTTTTGGCGGACGTGTATG  R: CAATCTCCTTCCTGCCTTTACC | 118 | 59.2 |
| GME7934_g | F: GGAAGAAACATCAAGCCAAGTGG  R: CATTCCCAGACCTCCACAACTC | 219 | 60.9 |
| GME9386_g | F: CCGTCACACCAATCAACTCTATC  R: CAACCTTGTAGAAATCAATCCCTG | 165 | 59.2 |
| GME8711_g | F: CTGGAAATGACCTGGCGTTATAC  R: TCTTGCTCACCCACATAAACACC | 205 | 60.2 |
| GME8678_g | F: GCACATGCAAACTGGGAAATCG  R: GATCCTGGTGTATCTCCTGTCG | 155 | 60.2 |
| GME1797_g | F: GATGATAGACTACGGCATGGATG  R: CACCCACCAATCTCCCAGTTAC | 144 | 61.0 |
| GME10493_g | F: GGGACCCTTTGATGTTGTTCTCC  R: CCGTTCTATCCTCGCTGATTCC | 103 | 61.8 |
| GME9838_g | F: CTGCTACTTCCGACTTGAGTGAC  R: CCTCCTCAATCTTGGGTCTATCC | 150 | 61.8 |
| GME9978_g | F: ATACGAGGTTCTTGTTGGCGAC  R: ATGAATGCACCATCGAGTTTCTC | 198 | 59.2 |
| GME6807_g | F: ACAAGTGCATCAAATTCCCCTG  R: TTCTTCTCCAGCCTTCCTCTTC | 156 | 59.2 |
| GME9454_g | F: CCCGTAGCAGAGGAGAAGTTTG  R: CCAGTCGCATTTATCGTAGGTTCC | 141 | 61.5 |
| GME10889_g | F: CCATCGACATCTGTGGGTATCC  R: TCAGACTCCTCATGCTCTCCTC | 172 | 61.5 |
| GME8409_g | F: GAGATGACGCAAAGCCAAGACC  R: TCACCACACTTGTTGCCCGAC | 136 | 61.5 |
| GME7725_g | F: AGTTGTGTGATGATGGCGGAG  R: TGAAAGCACCGTCCCTCTTACTC | 112 | 60.9 |
| GME8831_g | F: CCAGCAGCATCAAGTGATCTAC  R: AATGTCTACTCTGCACCTGGATC | 176 | 59.6 |
| GME6006_g | F: CATACAATGGCACTTCCGCTTG  R: CTTCTTCGTCCTCTTATGCTGTCC | 219 | 60.9 |
| GME1644_g | F: CTTACTACCCACCTCCCGATTTC  R: TCTGTTCTCTCGACCAACTCCTC | 130 | 61.8 |
| GME4506_g | F: GAAACCGCTTATCCCTGATGC  R: GATAATCCACCACTCCCACTTGC | 183 | 60.2 |
| GME4280_g | F: TATGCTTCCACCTCCCCAACC  R: GGTAACCATGCGCTCTTCTTGC | 109 | 61.5 |
| GME1998_g | F: ATCCCACCACCACTGTCGATTC  R: TATGATCCCCCTGAATACTGCG | 173 | 60.9 |
| GME1283_g | F: GGATAGAGGAGATGATCTGGACG  R: TTCATAACCACCCCGACTAGCAC | 127 | 61.8 |
| GME3988_g | F: CGAGATGAATACTATTGGCTCTGG  R: ACTCCGAATTGATACCCCTGCT | 179 | 59.6 |
| GAPDH | F: ATTGGCGTGGTGGTCGTAG  R: ACGGAAACATCAAGGGTAGGG | 142 | 59.6 |
