# Supplementary material for: Gene Expression Profiling Reveals Large Regulatory Switches between Succeeding Stipe Stages in Volvariella volvacea
Source: PLoS One. 2014 May 27;9(5):e97789. doi: 10.1371/journal.pone.0097789 (PMC4035324; doi:10.1371/journal.pone.0097789)
Supplement: Table S2 — Summary of the statistics of the digital gene expression data. (DOCX) [file pone.0097789.s004.docx]

Table S2. Summary of the statistics of the digital gene expression data

|  | BU | EG | EL | MA |
| --- | --- | --- | --- | --- |
| Raw tags | 5,779,135 | 6,110,156 | 6,087,503 | 6,079,153 |
| Clean tags | 5,612,361 | 5,978,522 | 5,972,497 | 5,967,968 |
| Proportion (clean tags/raw tags) | 97.11% | 97.85% | 98.11% | 98.17% |
| Distinct clean tags | 90,437 | 97,022 | 87,911 | 83,702 |
| Total of clean tags mapped to gene(s) | 1,234,056 | 1,614,697 | 1,541,433 | 1,914,000 |
| Total of clean tags mapped to genome | 3,541,886 | 3,573,977 | 3,641,203 | 3,446,455 |
| Total of unknown clean tags | 836,419 | 789,848 | 789,861 | 607,513 |
| Total of unambiguous clean tags mapped to gene(s) | 1,210,080 | 1,576,055 | 1,525,679 | 1,880,852 |
| Total of expressed genes | 6,972 | 7,355 | 6,558 | 6,834 |
